# Maternal Prepregnancy Body Mass Index, Gestational Weight Gain, and Risk of Adverse Perinatal Outcomes in Taiwan: A Population-Based Birth Cohort Study

**DOI:** 10.3390/ijerph17041221

**Published:** 2020-02-14

**Authors:** Chi-Nien Chen, Ho-Sheng Chen, Heng-Cheng Hsu

**Affiliations:** 1 Department of Pediatrics, National Taiwan University Hospital Hsinchu Branch, Hsinchu 30059, Taiwan; 2 Department of Pediatrics, National Taiwan University Children’s Hospital, Taipei 10041, Taiwan; draphy@gmail.com; 3 Department of Emergency, National Taiwan University Hospital, Taipei 10048, Taiwan; 4 Department of Obstetrics and Gynecology, National Taiwan University Hospital Hsinchu Branch, Hsinchu 30059, Taiwan; b101092037@gmail.com; 5 Department of Obstetrics and Gynecology, National Taiwan University Hospital, Taipei 10048, Taiwan

**Keywords:** obesity, gestational weight gain, preterm birth, perinatal complications, birth cohort

## Abstract

Epidemiological studies have shown that maternal prepregnancy body mass index (BMI) and gestational weight gain (GWG) are associated with increased risk of perinatal outcomes. However, the evidence of such associations in Asian populations is limited. We conducted a secondary data analysis to investigate the relationships of prepregnancy BMI and GWG with the risks of adverse perinatal outcomes, including gestational diabetes (GDM), gestational hypertension (GHTN), preeclampsia, cesarean delivery, preterm birth, low birth weight (LBW), and macrosomia. We categorized prepregnancy BMI by the WHO classification and GWG by the Institute of Medicine guidelines. We performed adjusted logistic regression models to estimate the odds ratios of adverse perinatal outcomes. A total of 19,052 women were included; prepregnancy overweight and obesity were associated with a greater risk of GDM, GHTN, preeclampsia, cesarean delivery, preterm birth, and macrosomia. Women with excessive GWG had a greater risk of GHTN, preeclampsia, cesarean delivery, and macrosomia. In conclusion, regardless of the range of GWG during pregnancy, maternal prepregnancy BMI is significantly associated with the risk of adverse perinatal outcomes in Taiwan. Public health attention regarding obesity reduction before conception and prenatal counseling for optimal GWG is needed to mitigate the risk of poor perinatal outcomes.

## 1. Introduction

Maternal mortality and morbidity remain significant public health concerns. Despite modern perinatal care, the rates of maternal pregnancy complications have continued to increase in recent decades [1]. The main risk factors for these poor pregnancy outcomes might contribute to chronic health conditions, including diabetes, hypertension, and cardiac disease [2]. Establishing a comprehensive perinatal care strategy to reduce the risk of certain adverse perinatal outcomes is imperative.

The prevalence of overweight and obesity among women at reproductive age is also increasing [3]. Obesity is another clinical concern and is linked to the development of the abovementioned chronic health conditions related to maternal morbidities. Maternal overweight and obesity are associated with an increased risk of many adverse perinatal outcomes, such as preterm birth, gestational diabetes, gestational hypertension (GHTN), preeclampsia, cesarean delivery and macrosomia [4,5,6,7,8,9,10,11,12]. However, the previous epidemiological studies are mainly from Western countries [4,5,6,7,8,9,10,11,12,13,14]. The prevalence of overweight and obesity is quite different among races and ethnicities, and there might be different effects on the risk of perinatal complications. The risk of preterm birth, being small for gestational age, cesarean delivery and macrosomia associated with maternal obesity may differ by race and ethnicity [14,15].

In addition to maternal obesity, gestational weight gain (GWG) has been found to be associated with the risk of poor maternal and neonatal outcomes [11,12,16,17]. Generally, clinicians utilize the Institute of Medicine (IOM) guidelines to educate pregnant women about the optimal GWG recommendations for different body mass index (BMI) categories [18]. While there have been published papers to provide GWG recommendations for Asian populations [19], a reliable guideline for GWG in Asian women is still lacking. Researchers still utilize IOM GWG recommendations as the main reference tool for Asian women [20,21]. There are differences in the range of GWG between Asian and White women [22]. The existing observational data are insufficient to determine whether these associations between GWG and adverse perinatal risks are similar in Asia, where the prevalence of overweight and obesity in women is much lower than in the United States [3,14]. Regarding heterogeneity by race and ethnicity, it is also questionable whether the IOM guidelines for GWG are reliable for the prediction of poor outcomes universally.

The development of specific recommendations for GWG for women of different races and ethnicities has previously been suggested [23]. Individual participant data meta-analysis studies already reported the impact of maternal prepregnancy BMI and GWG on perinatal complications [11,12], but important knowledge gaps remain to be clarified regarding the underlying mechanisms and whether the IOM guidelines are still applicable for predicting the risk of maternal and neonatal outcomes among women in countries with lower obesity rates. Only a few studies have tried to examine this association in Asian populations [14,16,24,25,26,27,28,29,30,31,32], and they were mainly hospital-based studies or conducted regionally within a single city. Further studies to determine whether the same findings could exist in population-based cohorts of different races and ethnicities are necessary. Therefore, the purpose of this study was to evaluate the association of prepregnancy BMI, gestational weight gain, and the risk of adverse perinatal outcomes by using a nationally representative birth cohort in Taiwan.

## 2. Methods

### 2.1. Study Participants

This is a secondary analysis of a prospective cohort derived from the Taiwan Birth Cohort Study (TBCS). The TBCS is a prospective, longitudinal cohort study with national representative samples of mother–infant dyads born between 1 January 2005 and 31 December 2005. The study participants were selected by a two-stage, stratified, systematic random sampling method to select participants from 369 cities and towns in Taiwan. TBCS was examining specifically neurocognitive development of infants in Taiwan, and to investigate the association between health conditions of infants, prenatal and postnatal factors, and the parents’ socioeconomic status. The detailed history and information of the TBCS have been previously described elsewhere [33,34].

Comprehensive face-to-face interviews and structured questionnaire surveys were conducted approximately 6 and 18 months after childbirth. In 2005, there were 206,741 live-born neonates in Taiwan, and a total of 24,200 mother–infant dyads (11.7% sampling rate) were enrolled initially. Participants with missing or invalid data and mothers with a major medical illness or pre-existing diabetes or multiple pregnancy were excluded. Data processing and analysis were conducted at the Health and Welfare Data Science Center between August 2018 and June 2019. This study was approved by the Institute Review Board (IRB) of National Taiwan University Hospital Hsin-Chu Branch (IRB No. 107-021-E).

### 2.2. Assessment of Body Mass Index and Gestational Weight Gain

Individual prepregnancy body mass index (BMI) was calculated from the reported data obtained in the questionnaire with the prepregnancy body weight and body height of mothers. All participants were categorized into four subgroups by the range of BMI according to the WHO classifications: underweight (BMI < 18.5 kg/m^2^), normal (BMI 18.5~25 kg/m^2^), overweight (BMI 25~30 kg/m^2^), and obese (BMI ≥ 30 kg/m^2^) [35]. The prepregnancy body weight of mothers was obtained from the response of the question “What was your last weight before this pregnancy?”, and the final body weight before delivery was obtained from the response of the question “What was your last weight before this delivery?”

GWG was defined as the difference between the final body weight before delivery and the prepregnancy body weight of mothers. GWG was further categorized into three subgroups according to the IOM guidelines of recommended weight gain during pregnancy as inadequate, within the range or excessive compared to the recommendations for different BMI categories (underweight: 12.5~18 kg; normal weight: 11.5~16 kg; overweight: 7–11.5 kg; obese 5~9 kg).

### 2.3. Outcome Assessments

The main outcomes were common adverse perinatal complications as follows: (1) gestational diabetes, (2) GHTN, (3) preeclampsia, (4) cesarean delivery, (5) preterm birth (gestational age less than 37 weeks), (6) low birth weight (LBW) infants (birth weight less than 2500 gm), and (7) macrosomia (birth weight more than 4000 gm). All outcome variables were obtained from the questionnaire answered by the mothers.

### 2.4. Covariates

The potential confounders associated with adverse perinatal outcomes were assessed for further analysis. Variables of parental childbearing age, parity, infant sex, smoking during pregnancy, maternal nationality, maternal educational status, family monthly income, and urbanicity of living area were collected from the TBCS databases. Parental childbearing age was categorized as “<25”, “25–29”, “30–34” and ≥35”. Parity was categorized as “primiparous” and “multiparous”. Maternal educational status was categorized as “below high school”, “high school”, and “college or above”. Family monthly income was categorized as “<30,000 New Taiwan Dollars (NTD)”, “30,000–50,000 NTD”, “50,000–70,000 NTD”, “70,000–100,000 NTD”, and “>100,000 NTD”. The urbanicity of residential area was categorized as “urban”, “town”, and “rural area”. Immigrant mothers were defined as mothers with nationalities of other countries.

### 2.5. Statistical Analysis

The clinical characteristics of the study participants in different ranges of BMI were compared by a chi-square test, Fisher’s exact test, or ANOVA. Participants in the normal BMI range were used as the reference group. We performed a logistic regression model to examine the risk of adverse perinatal complications associated with maternal prepregnancy BMI and GWG. Crude and adjusted regression models were performed, and subgroup analysis was performed to explore the interaction between prepregnancy BMI and GWG in terms of the main outcomes. The risk estimation is reported as an odds ratio (OR) with 95% confidence interval (95% CI). All statistical analyses were conducted using SAS Statistical Software version 9.4 (SAS Institute, Cary, NC, USA).

## 3. Results

The study sample included 21,248 women (87.8% of sampling cohort) who completed the interviews and questionnaire survey 6 months after childbirth. All participants had live-born children born in 2005. Participants with missing or invalid data, or who had a major medical illness, previous chronic hypertension, type 1 diabetes, or multiple births were excluded, and 19,052 women (89.7% of study samples) were enrolled for further investigation (Figure S1). The final cohort comprised 3851 women with underweight status (20.2%), 13,333 with normal status (70%), 1524 with overweight status (8%), and 344 with obese status (1.8%). The clinical characteristics of the final cohort are described in Table 1.

Table S1 summarizes the distribution of GWG among different prepregnancy BMI categories; 28.5% of participants had insufficient GWG, and 29.5% had excessive GWG. The incidence of adverse perinatal outcomes stratified by BMI categories is listed in Table 2. There were consistently increased prevalence rates of all adverse perinatal outcomes from underweight to obese status, except for the outcomes of preterm birth and LBW infants. The incidence of LBW infants in women with underweight status was the highest compared with the incidence associated with other categories. After controlling for the confounding factors, the adjusted odds ratios (aORs) for the risks of GDM, GHTN, preeclampsia, cesarean delivery, preterm birth, and macrosomia were significantly higher in women with prepregnancy overweight and obese status than in women with normal weight. The aOR for the risk of LBW infants was significantly higher in women with underweight status than in those with normal weight status (underweight: LBW: aOR = 1.55, 95% CI = 1.33–1.8).

Table 3 summarizes the association between perinatal outcomes and GWG. After controlling for confounding factors, the adjusted risks of GHTN, preeclampsia, cesarean delivery, and macrosomia were significantly higher in the excessive GWG group than in the adequate GWG group (excessive GWG: GHTN aOR = 2.51, 95% CI = 1.94–3.25; preeclampsia aOR = 3.17, 95% CI = 2.04–4.93; cesarean delivery aOR = 1.53, 95% CI = 1.42–1.65; macrosomia aOR = 2.66, 95% CI = 2.11–3.36). In contrast, the adjusted risks of preterm birth and LBW were significantly higher in the inadequate GWG group than in the adequate GWG group (inadequate GWG: preterm aOR = 1.67, 95% CI = 1.47–1.91; LBW aOR = 2.26, 95% CI = 1.95–2.62).

In the subgroup analysis, we divided GWG into three subgroups according to the IOM GWG guidelines to explore the effects of different GWGs on the association of prepregnancy BMI with perinatal complications (Table 4). The reference groups were normal weight women with inadequate, adequate, and excessive GWG. The results of the subgroup analysis showed that the risk of adverse perinatal outcomes, including GDM, GHTN, and preterm birth, were consistently significant in all the subgroup of excessive, adequate, and inadequate GWG in overweight and obese women; for the risk of cesarean delivery, mothers who were overweight and obese had significantly higher risk in adequate and excessive GWG group; for the risk of macrosomia, mothers who were overweight and obese in the excessive GWG group had significantly higher risk compared with the normal weight women.

## 4. Discussion

### 4.1. Main Findings

In this population-based birth cohort study, we demonstrated the low prevalence of overweight and obesity in women at reproductive age in Taiwan and explored the association between maternal prepregnancy BMI, GWG, and the risk of adverse perinatal outcomes. Our findings indicate that overweight and obesity are significantly associated with adverse perinatal outcomes, including GDM, GHTN, preeclampsia, preterm birth, cesarean delivery, and macrosomia. In a subgroup analysis, we found that the associations persisted in women with excessive GWG for the outcomes of GDM, GHTN, cesarean delivery, and preterm birth. Moreover, in overweight and obese women with inadequate GWG, the risks of GDM, GHTN and preterm birth were also prominent. Only underweight women had a greater risk of having LBW infants in the inadequate GWG subgroup.

The mechanisms behind the relationships between maternal obesity and adverse perinatal outcomes are complex, and several mechanisms might explain the associations [36]. Overweight and obesity are generally considered to be important risk factors for adverse maternal and neonatal outcomes due to the effects of oxidative stress [37], proinflammatory status [38], alterations in placental function [39,40], and insulin insensitivity [41]. Researchers in previous studies have suggested similar findings regarding this association. However, the effects of GWG on the development of adverse perinatal outcomes are also important for investigation to understand the underlying mechanisms of certain associations. Previous population cohort studies of the relationships between prepregnancy BMI and GWG and adverse outcomes were from Western countries [14]. Limited literature has focused on Asian populations. The prevalence of overweight and obesity is lower in Asia. Therefore, our results could provide more evidence on this topic and reveal differences across continents and ethnicities.

In a systematic review and meta-analysis study, Goldstein and colleagues reported that women in Western countries have a higher prepregnancy BMI than those in eastern Asia (China, Japan, Korea, and Taiwan) [14]. The IOM guidelines are mainly based on data from Western countries, and it may be inappropriate to apply the same recommendations in other regions, such as Asia, where the prevalence of overweight and obesity is lower than in the United States and Europe. However, there are no definite body weight gain references during pregnancy in Asian populations. We could only choose the IOM guidelines to evaluate the effects of GWG on the association.

Previous literature has revealed that women with high GWG have a lower risk of preterm birth and LBW infants [42]. Our study also confirmed these findings. Although the prevalence of overweight and obesity in our study sample was substantially lower than in the United States [3,14], the risks of preterm birth and LBW in women with high GWG remained significant. Our findings could provide evidence to verify the fact that the current IOM guidelines are suitable for clinicians to educate pregnant women regarding the optimal GWG for better maternal and neonatal outcomes.

### 4.2. Strengths and Limitations

The strength of our study is the large sample obtained from a nationally representative birth cohort in Taiwan. The study participants included selected samples from everywhere in Taiwan, not only from a single institute. We could perform a comprehensive investigation with sufficient large-scale samples to examine the association between prepregnancy BMI, GWG, and perinatal outcomes.

Our study had several limitations. First, the data of prepregnancy BMI and GWG were obtained and calculated based on self-reported questionnaires, and the adverse outcome variables were also obtained from self-reported questionnaires. There may be recall bias presented in the data analysis. Fortunately, previous studies showed that long-term maternal recall is accurate for prepregnancy height and weight [43], and GWG reported within one year postpartum may be reliable [44]. However, since the bias of misclassification of outcomes is usually toward the null, our study findings may underestimate the actual impact of maternal obesity and GWG [45,46]. Second, in the datasets provided by the Health and Welfare Science Center, we could access only restrictive datasets with categories of preterm or term infants, but not the full authorized primary gestational age data to evaluate and differentiate the risk of severity of prematurity in terms of late preterm or extremely preterm. Additionally, we could not assess the risk of small for gestational age or large for gestational age as in a previous study [16]. Third, the data were from 2005, and may not fully reflect the current situation. Fourth, it is unfair to compare GWG in cases of preterm birth with the GWG of term pregnancies. The weight gain might be lower for those with premature delivery. In our study, we also performed subgroup analysis to evaluate the effect of GWG on preterm risk. For women with overweight and obesity, the risk of preterm birth was still significantly higher in the subgroup with insufficient GWG. The effects of GWG may act as a mediator, as discussed in a previous publication [47]. Additionally, the effect of prepregnancy BMI status may influence the development of preterm birth.

In 2004, a recommended BMI cut-off point to define overweight and obesity in Asian populations was suggested by WHO expert consultation to better predict adverse health outcomes [48]. However, a universal BMI cut-off point may not be appropriate for all Asian populations, for example, Mongolians may have a greater rate of obesity than Japanese [49]. In Canada, Asian women also have a greater risk of inadequate GWG and reduced risk of excessive GWG compared with White and Black women by the WHO BMI criteria for obesity and IOM GWG recommendation [20]. The heterogeneity of race and ethnicity may be the key point to be discussed for the prediction of adverse outcomes in different regional or global recommendations and guidelines.

Since maternal prepregnancy overweight and obesity and inadequate or excessive GWG are significantly associated with the risk of several adverse perinatal outcomes, aggressive management to improve both maternal and neonatal health is important. Prepregnancy weight loss with a 10% BMI reduction may contribute to a 10% lower risk of pregnancy outcomes [50]. Lifestyle interventions are common strategies to reduce GWG in overweight and obese women during pregnancy. However, in some meta-analysis and randomized control studies, the investigators reported that lifestyle interventions had little effect on the adverse outcomes, although they could reduce GWG during pregnancy [51,52,53]. Therefore, we should place greater emphasize on both the management of obesity among women during preconceptional periods and aggressive lifestyle intervention at the early pregnancy stage to maintain normal GWG. Further studies of the efficient management of maternal obesity should be performed to mitigate the risk of these adverse perinatal outcomes. Nutrition support during pregnancy is also important for mothers and children [54]. Clinicians should provide prenatal counseling to these high-risk women about dietary intake and physical activity to prevent unexpected weight gain [55]. In addition, because of the heterogeneity of race and ethnicity, the recommendation for GWG may be different across continents. Establishing a new guideline and GWG recommendation for Asian populations is necessary for optimal risk reduction during pregnancy. Our findings have clinical practice and public health implications regarding improving maternal and neonatal outcomes.

## 5. Conclusions

In conclusion, a prepregnancy BMI that indicates overweight and obesity or excessive GWG may cause adverse perinatal outcomes. Our research could be used to establish potential guidelines for GWG for better maternal and neonatal outcomes. Further investigations are warranted to establish a specific GWG recommendation according to the heterogeneity of race and ethnicity.

## Figures and Tables

**Table 1:** Clinical characteristics of the study population.

	Total	Underweight	Normal	Overweight	Obese
	*N* = 19,052	*N* = 3851	*N* = 13,333	*N* = 1524	*N* = 344
Prepregnancy BMI *	20.31	17.74	20.7	26.67	32.03
Maternal age *					
<25	4323 (22.7)	1313 (34.1)	2697 (20.2)	251 (16.5)	62 (18)
25–29	7211 (37.9)	1478 (38.4)	5055 (37.9)	543 (35.6)	135 (39.3)
30–34	5527 (29)	825 (21.4)	4098 (30.7)	500 (32.8)	104 (30.2)
≥35	1991 (10.4)	235 (6.1)	1483 (11.1)	230 (15.1)	43 (12.5)
Parity *					
primiparous	9771 (51.3)	2391 (62.1)	6703 (50.3)	547 (35.9)	130 (37.8)
multiparous	9281 (48.7)	1460 (37.9)	6630 (49.7)	977 (64.1)	214 (62.2)
Infant sex *					
Male	9996 (52.5)	1967 (51.1)	7053 (52.9)	789 (51.8)	187 (54.4)
Maternal education status *					
<high school	3270 (17.2)	832 (21.6)	2149 (16.1)	220 (14.5)	69 (20.1)
High school	11,881 (62.4)	2395 (62.2)	8177 (61.3)	1072 (70.3)	237 (68.9)
Above college	3901 (20.4)	624 (16.2)	3007 (22.6)	232 (15.2)	38 (11)
Family monthly income *					
<30,000 NTD	2164 (11.3)	522 (13.5)	1426 (10.7)	167 (11)	49 (14.2)
3–50,000 NTD	5743 (30.1)	1224 (31.8)	3840 (28.8)	542 (35.6)	137 (39.8)
5–70,000 NTD	4982 (26.2)	1019 (26.5)	3420 (25.6)	445 (29.2)	98 (28.5)
7–100,000 NTD	4048 (21.3)	700 (18.2)	3034 (22.8)	273 (17.9)	41 (11.9)
≥100,000 NTD	2115 (11.1)	386 (10)	1613 (12.1)	97 (6.3)	19 (5.5)
Urbanicity *					
Urban	9023 (47.4)	1836 (47.7)	6388 (47.9)	652 (42.8)	147 (42.7)
Town	5287 (27.8)	1047 (27.2)	3686 (27.7)	456 (29.9)	98 (28.5)
Rural	4742 (24.8)	968 (25.1)	3259 (24.4)	416 (27.3)	99 (28.8)
Smoking during pregnancy *					
Yes	629 (3.3)	178 (4.6)	377 (2.8)	57 (3.7)	17 (4.9)
Immigrant mothers *					
Yes	2474 (13)	710 (18.4)	1693 (12.7)	65 (4.3)	6 (1.7)

Data are presented as number (%) or median. * *p*-value < 0.05 by chi-square test, Fisher’s exact test, or ANOVA.

**Table 2:** Association between maternal prepregnancy body mass index (BMI) status and perinatal outcomes.

	BMI Status	Case	%	cOR	95% CI	aOR *	95% CI
**GDM**	Underweight	40	1.04	0.55	0.39–0.76	0.64	0.46–0.9
	Normal	252	1.89	1	Reference	1	Reference
	Overweight	69	4.53	2.46	1.88–3.23	2.44	1.84–3.22
	Obese	21	6.1	3.38	2.13–5.34	3.54	2.21–5.65
**GHTN**	Underweight	23	0.6	0.47	0.3–0.73	0.49	0.32–0.76
	Normal	169	1.27	1	Reference	1	Reference
	Overweight	68	4.46	3.64	2.73–4.85	3.72	2.77–4.98
	Obese	37	10.76	9.39	6.46–13.64	9.68	6.59–14.24
**Preeclampsia**	Underweight	8	0.21	0.37	0.18–0.77	0.37	0.18–0.77
	Normal	74	0.56	1	Reference	1	Reference
	Overweight	16	1.05	1.9	1.11–3.27	1.87	1.08–3.26
	Obese	10	2.91	5.37	2.75–10.48	5.01	2.53–9.93
**CS**	Underweight	1025	26.62	0.78	0.72–0.84	0.86	0.79–0.93
	Normal	4247	31.85	1	Reference	1	Reference
	Overweight	666	43.7	1.66	1.49–1.85	1.57	1.41–1.76
	Obese	194	56.4	2.76	2.23–3.43	2.7	2.16–3.36
**Preterm birth**	Underweight	252	6.54	1.06	0.92–1.23	1.12	0.97–1.3
	Normal	826	6.2	1	Reference	1	Reference
	Overweight	146	9.58	1.6	1.33-1.93	1.47	1.22–1.77
	Obese	40	11.63	1.99	1.42-2.79	1.76	1.25–2.48
**LBW**	Underweight	271	7.04	1.61	1.39–1.87	1.55	1.33–1.8
	Normal	598	4.49	1	Reference	1	Reference
	Overweight	82	5.38	1.21	0.96–1.54	1.16	0.91–1.47
	Obese	18	5.23	1.18	0.73–1.9	1.06	0.66–1.73
**Macrosomia**	Underweight	32	0.83	0.45	0.31–0.65	0.47	0.32–0.68
	Normal	245	1.84	1	Reference	1	Reference
	Overweight	71	4.66	2.61	1.99–3.42	2.72	2.06–3.59
	Obese	18	5.23	2.95	1.81–4.82	3.16	1.92–5.21

* Adjusted for maternal age, maternal immigration status, parity, maternal smoking during pregnancy, family income, maternal educational status, infant sex, living area. Abbreviations: CI, confidence interval; GDM, gestational diabetes; GHTN, gestational hypertension; CS, cesarean section; LBW, low birth weight; cOR, crude odds ratio; aOR, adjusted odds ratio.

**Table 3:** Association between perinatal outcomes and gestational weight gain.

	GWG	Case	%	cOR	95% CI	aOR *	95% CI
**GDM**	Inadequate	104	1.91	1.03	0.8–1.32	1.1	0.85–1.41
	Adequate	149	1.86	1	Reference	1	Reference
	Excessive	129	2.29	1.24	0.97–1.57	1.27	0.99–1.62
**GHTN**	Inadequate	41	0.75	0.65	0.45–0.94	0.69	0.47–0.99
	Adequate	93	1.16	1	Reference	1	Reference
	Excessive	163	2.9	2.54	1.96–3.28	2.51	1.94–3.25
**Preeclampsia**	Inadequate	11	0.2	0.56	0.28–1.12	0.59	0.29–1.18
	Adequate	29	0.36	1	Reference	1	Reference
	Excessive	68	1.21	3.36	2.17–5.2	3.17	2.04–4.93
**CS**	Inadequate	1529	28.14	0.92	0.85–0.99	0.95	0.87–1.02
	Adequate	2395	29.96	1	Reference	1	Reference
	Excessive	2208	39.25	1.51	1.41–1.62	1.53	1.42–1.65
**Preterm**	Inadequate	522	9.61	1.71	1.5–1.94	1.67	1.47–1.91
	Adequate	469	5.87	1	Reference	1	Reference
	Excessive	273	4.85	0.82	0.7–0.95	0.8	0.69–0.94
**LBW**	Inadequate	459	8.45	2.18	1.88–2.52	2.26	1.95–2.62
	Adequate	325	4.07	1	Reference	1	Reference
	Excessive	185	3.29	0.8	0.67–0.96	0.74	0.62–0.89
**Macrosomia**	Inadequate	47	0.87	0.6	0.43–0.84	0.59	0.42–0.83
	Adequate	115	1.44	1	Reference	1	Reference
	Excessive	204	3.63	2.58	2.05–3.25	2.66	2.11–3.36

*Adjusted for maternal age, infant sex, parity, maternal education, maternal immigration status, family monthly income, urbanicity of living area, and smoking during pregnancy. Abbreviations: GWG, gestational weight gain; GDM, gestational diabetes; GHTN, gestational hypertension; CS, cesarean section; LBW, low birth weight; cOR, crude odds ratio; aOR, adjusted odds ratio.

**Table 4:** Subgroup analysis of the association between prepregnancy body mass index (BMI) and risk of adverse perinatal outcomes by gestational weight gain categories.

		Underweight		Overweight		Obese	
Outcomes	GWG	aOR *	95% CI	aOR *	95% CI	aOR *	95% CI
**GDM**	Inadequate	0.62	0.36–1.09	2.48	1.2–5.16	4.01	1.17–13.83
	Adequate	0.55	0.31–0.96	2.3	1.43–3.69	3.53	1.48–8.43
	Excessive	0.87	0.44–1.71	2.76	1.82–4.2	3.53	1.85–6.73
**GHTN**	Inadequate	0.5	0.19–1.32	4.2	1.55–11.4	13.58	4.33–42.61
	Adequate	0.37	0.16–0.86	3.24	1.85–5.69	10.67	4.95–23.02
	Excessive	0.83	0.45–1.54	2.98	2.04–4.36	6.2	3.78–10.15
**Preeclampsia**	Inadequate	NA	-	2.14	0.24–19.46	21.35	4–113.87
	Adequate	0.42	0.12–1.42	2.22	0.74–6.62	NA	-
	Excessive	0.66	0.26–1.69	1.22	0.62–2.4	3.3	1.5–7.27
**CS**	Inadequate	1.03	0.89–1.18	1.36	0.99–1.87	2.87	1.48–5.58
	Adequate	0.86	0.75–0.97	1.49	1.24–1.79	2.65	1.76-3.99
	Excessive	0.85	0.71–1.01	1.42	1.21–1.66	2.21	1.66–2.95
**Preterm birth**	Inadequate	1.15	0.93–1.42	2.18	1.47–3.24	2.97	1.38–6.37
	Adequate	0.84	0.65–1.09	1.77	1.31–2.4	2.05	1.1–3.81
	Excessive	1.06	0.71–1.57	1.49	1.08–2.06	1.95	1.17–3.23
**LBW**	Inadequate	1.67	1.35–2.05	2.41	1.57–3.69	1.59	0.56–4.54
	Adequate	1.13	0.86–1.49	1.32	0.87–2	1.92	0.87–4.23
	Excessive	0.99	0.64–1.57	0.95	0.62–1.48	0.9	0.41–1.97
**Macrosomia**	Inadequate	0.58	0.25–1.32	1.78	0.53–5.95	6.04	1.37–26.71
	Adequate	0.63	0.35–1.14	2.06	1.2–3.55	2.19	0.67–7.19
	Excessive	0.52	0.28–0.94	2.22	1.57–3.13	2.14	1.17–3.9

* Adjusted for maternal age, infant sex, parity, maternal education, maternal immigration status, family monthly income, urbanicity of living area, and smoking during pregnancy. The reference groups were normal weight women with inadequate, adequate, and excessive GWG. Abbreviation: GDM, gestational diabetes; GHTN, gestational hypertension; CS, cesarean section; LBW, low birth weight; aOR, adjusted odds ratio. NA: not available. Some of the odds ratios could not be calculated due to the low frequency of certain outcomes.

## Supplementary Materials

The following are available online at https://www.mdpi.com/1660-4601/17/4/1221/s1, Figure S1: Flow chart of the study population. Table S1: Distribution of gestational weight gain among different BMI statuses.
