# Supplementary material for: Maternal Prepregnancy Body Mass Index, Gestational Weight Gain, and Risk of Adverse Perinatal Outcomes in Taiwan: A Population-Based Birth Cohort Study
Source: Int J Environ Res Public Health. 2020 Feb 14;17(4):1221. doi: 10.3390/ijerph17041221 (PMC7068269; doi:10.3390/ijerph17041221)
Supplement: Supplementary file 1 [file ijerph-17-01221-s001.pdf]

Supplementary materials:

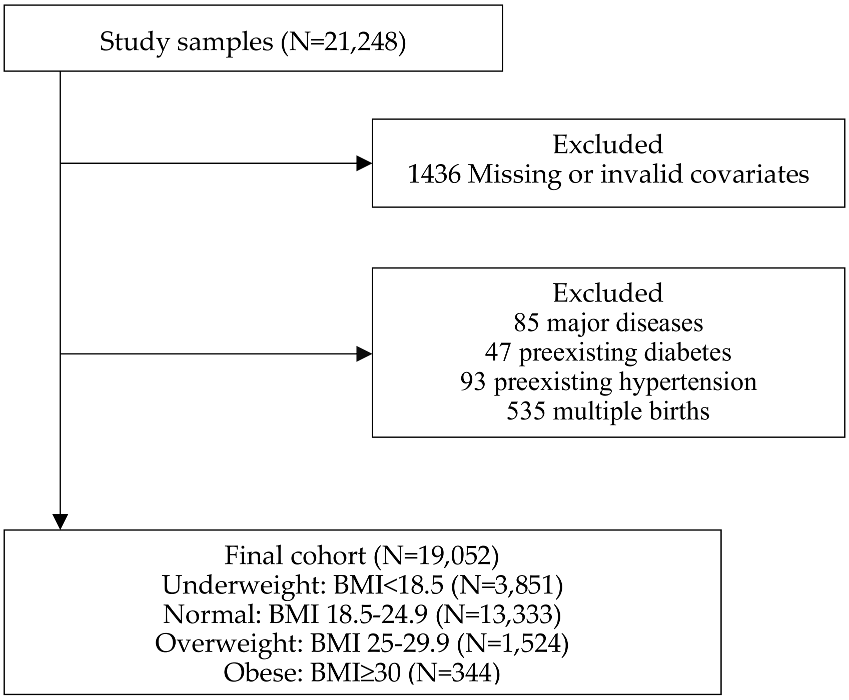

Figure S1. Flow chart of the study population.

Table S1. Distribution of gestational weight gain among different BMI statuses.

	Mean	SD	Median	Percentile				GWG by IOM guideline		
				10	25	75	90	Insufficient, n (%)	Adequate, n (%)	Excessive, n (%)
Total (N=19,052)	14.16	5.01	14	8	11	17	20	5433 (28.52)	7994 (41.96)	5625 (29.52)
Underweight (N=3,851)	14.36	4.8	14	9	11	17	20.5	1479 (38.41)	1668 (43.31)	704 (18.28)
Normal (N=13,333)	14.36	4.92	14	8.6	11	17	20.5	3739 (28.04)	5674 (42.56)	3920 (29.4)
Overweight (N=1,524)	12.52	5.62	12	6	9	15.9	20	178 (11.68)	554 (36.35)	792 (51.97)
Obese (N=344)	11.23	5.89	10.6	4	7	14	19	37 (10.76)	98 (28.49)	209 (60.76)
